# Systematic review and feasibility study on pre-analytical factors and genomic analyses on archival formalin-fixed paraffin-embedded breast cancer tissue

**DOI:** 10.1038/s41598-024-69285-8

**Published:** 2024-08-06

**Authors:** Dimitrios Salgkamis, Emmanouil G. Sifakis, Susanne Agartz, Valtteri Wirta, Johan Hartman, Jonas Bergh, Theodoros Foukakis, Alexios Matikas, Ioannis Zerdes

**Affiliations:** 1 Department of Oncology-Pathology, Karolinska Institutet, Stockholm, Sweden; 2 Department of Microbiology, Tumor and Cell Biology, Clinical Genomics Stockholm, Science for Life Laboratory, Karolinska Institutet, Stockholm, Sweden; 3 Department of Clinical Pathology and Cancer Diagnostics, Karolinska University Hospital, Stockholm, Sweden; 4 Breast Center, Theme Cancer, Karolinska University Hospital, Stockholm, Sweden; 5 Theme Cancer, Karolinska University Hospital, Stockholm, Sweden

**Keywords:** Formalin-fixed Paraffin-embedded, Preanalytical factors, Next-generation sequencing, Gene expression, Breast cancer, Computational biology and bioinformatics, Medical research, Oncology, Breast cancer, Cancer, Cancer genomics, Clinical genetics, Gene expression

## Abstract

Formalin-fixed paraffin-embedded (FFPE) tissue represents a valuable source for translational cancer research. However, the widespread application of various downstream methods remains challenging. Here, we aimed to assess the feasibility of a genomic and gene expression analysis workflow using FFPE breast cancer (BC) tissue. We conducted a systematic literature review for the assessment of concordance between FFPE and fresh-frozen matched tissue samples derived from patients with BC for DNA and RNA downstream applications. The analytical performance of three different nucleic acid extraction kits on FFPE BC clinical samples was compared. We also applied a newly developed targeted DNA Next-Generation Sequencing (NGS) 370-gene panel and the nCounter BC360® platform on simultaneously extracted DNA and RNA, respectively, using FFPE tissue from a phase II clinical trial. Of the 3701 initial search results, 40 articles were included in the systematic review. High degree of concordance was observed in various downstream application platforms. Moreover, the performance of simultaneous DNA/RNA extraction kit was demonstrated with targeted DNA NGS and gene expression profiling. Exclusion of variants below 5% variant allele frequency was essential to overcome FFPE-induced artefacts. Targeted genomic analyses were feasible in simultaneously extracted DNA/RNA from FFPE material, providing insights for their implementation in clinical trials/cohorts.

## Introduction

Breast cancer (BC) represents a heterogeneous disease both clinically and biologically. Gene sequencing and gene expression profiling have emerged as important pillars in diagnostics and disease classification, biomarker discovery and treatment strategies in early and metastatic BC^1^. For such studies, archival formalin-fixed paraffin-embedded (FFPE) remains one of the main valuable patient tissue material sources for translational research and/or clinical applications and often is the only available patient tissue source. However, the use of nucleic acids derived from FFPE material for downstream applications has been challenging mainly due to pre-analytical setbacks, including tissue handling, extraction protocol, storage conditions, age, biospecimen size, formalin composition, delay of fixation and time in fixative^2^.

Given that the isolated nucleic acids are prone to degradation^3,4^, proper preservation of the biospecimen is of utmost importance to prevent risk of contamination, cell degeneration and nucleic acid degradation^5^. The most cost-efficient method for prolonged storage of clinical samples for a long time period, at ambient temperature and nonsterile conditions remains the use of formalin^6^. During fixation, a cross-linking occurs between formaldehyde and proteins, where formaldehyde triggers the formation of stable methylene bridges, thus making the tissue harsher^7^. Considering that nucleic acids are generally degraded due to the cross-linking and fixation process, FFPE tissue could therefore be less optimal for molecular downstream applications compared to fresh-frozen (FF) tissue^8–10^. Another factor that can affect the yield and the quality of the nucleic acids isolated from FFPE material is the extraction method. Several commercially available extraction kits offer robust performance but lack analytical validity and optimisation in different disease settings^11^. Thus, improving the yield and quality of nucleic acids extracted from FFPE becomes a pertinent issue to facilitate costly downstream analyses.

In this study, we aimed (i) to evaluate the degree of concordance between FFPE and FF material in matched BC tissue samples for certain RNA and DNA applications in published literature, (ii) to demonstrate the feasibility of simultaneous DNA and RNA extraction method on FFPE material using commercially available kits and (iii) to explore its performance on the hybridization-based nCounter^®^ platform for gene expression analysis and on an in-house designed targeted NGS panel for genomic analysis using archival material from a randomized phase II early BC clinical trial.

## Material and methods

### Systematic literature search, inclusion criteria, data extraction and analysis

In order to identify studies evaluating the degree of concordance of the performance of downstream applications on the extracted nucleic acids between FF and FFPE matched BC tissue material, we conducted a systematic literature review in accordance with the PRISMA 2020 statement^12^. The systematic electronic search was performed in the following databases: Medline (Ovid), Embase, Web of Science (Clarivate), and PubMed Central. The original search was performed by two librarians at the Karolinska Institutet University Library on February 5th 2021 and the search was updated on February 2nd 2023 using the methods described by Bramer and Bain^13^. The search strategy was developed in Medline (Ovid). For each search concept, Medical Subject Headings (MeSH-terms) and free-text terms were identified. The search was then translated into the other databases. Language was restricted to English and databases were searched from inception. The strategies were peer-reviewed by another librarian prior to execution. De-duplication was performed as previously described^14^. The full search strategies for all databases are available as Supplementary Material S1. Three additional sources were used to ensure that all relevant articles were included: (i) the references of selected review articles on the topic were reviewed; (ii) secondary referencing by manually reviewing reference lists of potentially eligible articles; (iii) the Biospecimen Research Database (BRD) [http://biospecimens.cancer.gov/brd. Bethesda (MD), National Cancer Institute]. Studies were included in the systematic review only if they fulfilled the following criteria: studies that used nucleic acids (DNA and/or RNA) extracted from FF and FFPE matched tissue samples, derived from patients with BC, and applied certain technology platforms or methods i.e. microarray-based/DASL (cDNA-mediated annealing, extension, selection and ligation assay), multiplex hybridization nCounter^®^, RNA-sequencing technology platforms for RNA and Sanger and Next-Generation Sequencing for DNA. Case reports, reviews, prior systematic reviews, animal studies, conference material, editorials, letters and notes were excluded. Data extraction was performed using a predefined form for each study including the following variables: first author’s name, name of the journal, date of publication, type of nucleic acid (DNA/RNA); number of matched FF-FFPE tissue samples derived from patients with BC, degree of concordance reported as coefficient of determination ($R^{2}$), Spearman and/or Pearson correlation coefficients or Lin’s concordance correlation coefficient, or descriptively as reported by the authors when the same technology platform and analysis was performed using nucleic acids extracted from matched FF-FFPE BC tissue material. The title and abstract screening, full-text screening, and data extraction were performed by two investigators (D.S., I.Z.) and any discrepancies were resolved by a third investigator (A.M.).

### Patient material

Archival surgical FFPE tissue material was used from thirty patients enrolled in the Scandinavian Breast Group (SBG) 2004-1 multicenter randomized phase II clinical trial. The trial enrolled a total of 124 patients with high-risk early BC and evaluated the feasibility of three adjuvant chemotherapy regimens: dose-dense epirubicin/cyclophosphamide (EC) followed by dose-dense docetaxel; the same regimen with additional tailored dosing according to hematologic toxicity during the previous treatment cycle; or concomitant docetaxel, doxorubicin and cyclophosphamide. The study design, feasibility and short-term toxicity^15^, long-term efficacy^16^ and immunogenomic analyses based on tissue material from the study^17^ have been previously reported. This trial was initiated in 2004, when trial registration was not compulsory. It is the feasibility study of the randomized phase III trial (PANTHER^18^, EudraCT number 2007-002061-12 and Clinicaltrials.gov accession number NCT00798070). All correlative analyses for this clinical trial have been approved by the Ethics Committee at Karolinska Institutet (Dnr 2017/345-32 and Dnr 2018/1084-32). In addition, anonymized archival surgical FFPE tissue material from patients with primary breast cancer (dated from 1992 to 2015) was used to assess the analytical performance of different commercially available DNA and RNA extraction kits, as described hereunder.

### FFPE tissue preparation, block annotation and sectioning

Surgical FFPE BC tissue blocks were preserved at 4 $^\circ$C, after tissue fixation with 10% neutral-buffered formalin and paraffin embedment. Hematoxylin and eosin-stained sections 4 $\upmu$m in thickness from each FFPE BC tissue block were obtained and tumour-rich areas were annotated by a certified pathologist (J.H.). The tumour-rich blocks were subsequently sectioned (thickness: 10 $\upmu$m), using the Epredia^™^ HM 355S Automatic Microtome (Thermo Fisher Scientific, USA) and stored at −20 $^\circ$C until nucleic acid isolation.

### Nucleic acid extraction from FFPE BC tissue samples

Tumour-rich surgical FFPE BC tissue blocks were cut into sections, each of 10 $\upmu$m in thickness. Two consecutively cut sections were used as starting material for each extraction protocol kit. The sections were then deparaffinized using xylene prior the purification of DNA and/or RNA. DNA extraction was performed using the QIAamp DNA FFPE Tissue kit (Cat No 56404, QIAGEN, Germany) and the AllPrep DNA/RNA FFPE kit (Cat No 80234, QIAGEN, Germany) according to the manufacturer’s instructions. However, DNA was eluted in Buffer EB (Cat No 19086, QIAGEN, Germany) instead of EDTA-containing Buffer ATE in order to prevent any enzymatic inhibition. Total RNA was extracted using the RNeasy FFPE kit (Cat No 73504, QIAGEN, Germany) and the AllPrep DNA/RNA FFPE kit, according to the manufacturer’s instructions (by also including small RNAs) and eluted in RNase-free water. To minimize the risk of any potential contamination in the RNA purification, DNase I digestion step was performed using the RNase-free DNase set (Cat No 79254, QIAGEN, Germany). For DNA purification, RNase A (100 mg/mL) (Cat No 19101, QIAGEN, Germany) step was performed accordingly. All nucleic acids were collected in Eppendorf^®^ Forensic DNA Grade Safe-Lock 1.5 mL microcentrifuge tubes (Eppendorf SE, Germany), upon double elution at 20,000*g*, and stored at − 80 $^\circ$C. For every patient with BC, tumour DNA and RNA were extracted from two tumour-rich surgical FFPE tissue sections using only the AllPrep DNA/RNA FFPE kit. Tumour DNA and RNA were extracted from thirty patients enrolled in the SBG 2004-1 study using only the AllPrep DNA/RNA FFPE kit, while matched germline DNA was extracted from frozen whole peripheral blood, previously stored in EDTA-tubes at − 80 $^\circ$C, using FlexiGene DNA kit (Cat No 51206, QIAGEN, Germany) according to the manufacturer’s instructions.

### Quality control and nucleic acid yield estimation

The initial quality control (QC) included: (i) the Nanodrop^™^ ND-1000 (Saveen Werner, Sweden), ultraviolet spectrophotometer to assess the purity^19^ of DNA and RNA samples based on the absorbance maximum at 260 nm ($A_{260}$) for nucleic acids, at 280 nm ($A_{280}$) for proteins, at 230 nm ($A_{230}$) for organic and salt isolation compounds, (ii) the Qubit^®^ 3.0 Fluorometer (Thermo Fisher Scientific, USA) as well as the Qubit^™^ dsDNA BR Assay Kit (Cat No Q32850, Invitrogen, USA) and Qubit^™^ RNA BR Assay Kit (Cat No Q10210) to quantify the DNA and the RNA samples, respectively, and (iii) the automated electrophoresis 2200 Tapestation^®^ System (Agilent Technologies, Santa Clara, CA, USA) and the Genomic DNA (Cat No 5067–5365) and RNA (Cat No 5067–5576) ScreenTapes to estimate the DNA Integrity Number (DIN) for DNA samples and RNA Integrity Number equivalent (RIN$^{e}$) for RNA samples, respectively. An additional quality metric DV$_{200}$ for the RNA samples was generated manually, by using the raw data as presented in the region table in TapeStation Analysis Software, as follows: DV$_{200}$ = (Number of RNA fragments with sizes between 200 nt and 10,000 nt / Total number of RNA fragments) * 100. The yield comparison of RNA extraction kits (RNeasy and AllPrep) was done after performing paired t-test for the RNA samples extracted from 7 FFPE blocks. Similarly, the yield comparison of DNA extraction kits (AllPrep and QIAamp) was done after performing paired t-test for the DNA samples extracted from 5 FFPE blocks.

### Targeted DNA sequencing and bioinformatics analysis

Tumour DNA and matched germline DNA samples derived from 30 patients with breast cancer enrolled in SBG2004-1 clinical trial were sequenced using a newly designed targeted DNA panel. This custom-developed DNA panel consisting of 370 genes (also referred to as GMCK Solid Cancer Panel) was used within the NovaSeq^™^ 6000 system (Illumina Inc.) with a 1000x average coverage. This targeted DNA GMCK Solid Cancer Panel (v1.0), 2.4 Mb in total size, was designed using the reference genome hg19, providing the rationale for identification of somatic short variants (Single-nucleotide Polymorphisms and Short Insertions and Deletions, SNVs/INDELs), Copy-number Alterations (CNAs), fusion events, Microsatellite Instability (MSI) and estimation of the Tumour Mutational Burden (TMB). Detailed description of the GMCK Solid Cancer Panel v1.0 panel is provided as Supplementary Material S1.

The bioinformatics analysis can be summarized as follows. Pre-processing following BALSAMIC workflow v9.0.1^20^ was used to analyze each of the FASTQ files. Firstly, a quality control of FASTQ files using FastQC v0.11.9 was performed^21^. Adapter sequences and low quality bases were trimmed using fastp v0.23.2^22^. Trimmed reads were mapped to the reference genome hg19 using BWA MEM v0.7.17^23^. The resulted SAM files were converted to BAM files and sorted using samtools v1.15.1^24,25^. Duplicated reads were marked using Picard tools MarkDuplicates v2.27.1^26^ and promptly quality controlled using CollectHsMetrics, CollectInsertSizeMetrics, and CollectAligntmentSummaryMetrics functionalities. Results of the quality controlled steps were summarized by MultiQC v1.12^27^. For each sample, somatic mutations were called using VarDict v2019.06.04^28^ in tumour-normal mode and annotated using Ensembl VEP v104.3^29^. Apart from VarDict’s internal filters to report the variants, the called variants were also further post-filtered based on depth, frequency and quality, according to two filters followed in Blue Collar Bioinformatics (bcbio-nextgen) https://zenodo.org/records/5781867. Specifically, the first filter looks at regions with low depth for allele frequency (AF * DP < 6), and within these calls, it filters if a call has low mapping quality and multiple mismatches in a read ((AF * DP < 6) && ((MQ < 55.0 && NM > 1.0) || (MQ < 60.0 && NM > 2.0) || (DP < 10) || (QUAL < 45))). The second one filters in low allele frequency regions with poor quality if all of these are true: ((AF < 0.2) & (QUAL < 55) & (SSF > 0.06)). Only those variants that fulfilled the filtering criteria and scored as PASS in the VCF file were reported. Due to previously reported FFPE representative, artefactual C-T conversions that result from cytosine deamination during formalin fixation^30,31^ an additional filtering criterion was applied to exclude variants below 5% variant allele frequency^32^. Different post-filtering strategies could also be applied, e.g., different quality criteria or utilizing population databases, like the gnomAD database^33^ and the COSMIC somatic mutation database^34^, but this goes beyond the scope of the current feasibility study.

Mutational spectrums were identified using the R package MutationalPatterns v.1.2.1^35^, and de-novo signatures were extracted based on the non-negative matrix factorization (NMF) algorithm.

### nCounter^®^ Breast Cancer 360^™^ Panel

RNA extracted from two patients with BC, previously enrolled in the SBG2004-1 clinical trial was used for expression profiling 776 gene targets using the nCounter^®^ Breast Cancer 360^™^ gene panel (Nanostring Technologies, Seattle, USA) according to manufacturer’s instructions. Both the quality control and data pre-processing were performed in the nSolver Analysis Software version 4.0 (Nanostring Technologies, Seattle, USA). The raw data of the assay were assessed using quality assurance metrics to measure imaging quality, oversaturation, and overall signal-to-noise ratio. Background thresholding was performed with the default cut-off value of 20. The background-corrected data were then normalized using the geometric mean for two normalization factors, i.e., the 6 positive controls and the 18 housekeeping genes included in the assay.

## Results

### Literature search and study characteristics

**Figure 1:**
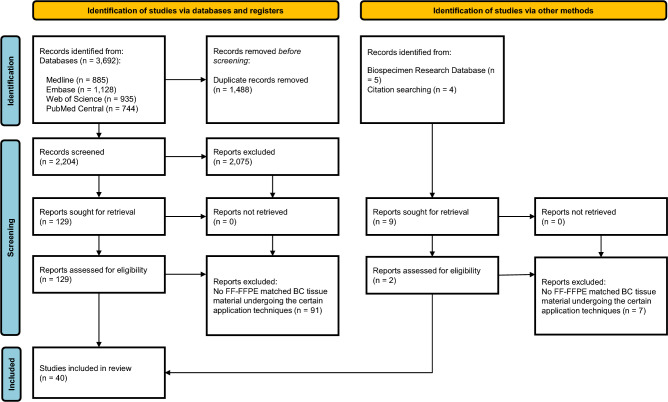
PRISMA 2020 flow diagram.

A total of 3692 records were identified from the four databases (Medline, Embase, Web of Science and PubMed Central) and 9 records were identified via other methods (5 from Biospecimen Research Database and 4 from citation searching). Upon de-duplication, 2204 records identified from Databases, 131 were retrieved for full-text review and 40 articles were included in the review. The flowchart of study selection is presented in Fig. 1. Among the selected studies using matched FF-FFPE BC tissue samples, 31 articles evaluated only the performance of RNA, 8 articles only the performance of DNA and one article reported the performance of both RNA and DNA. All studies are presented in Tables 1 and 2, respectively.

Among the 32 studies that explored the performance of RNA extracted from matched FF-FFPE BC tissue material (Table 1) the degree of concordance was not reported in only 5 studies. Five studies used cDNA-mediated Annealing, extension, Selection and Ligation assay (DASL) while 9 studies used other microarray-based applications and reported varying concordance. Hybridization-based assays were used in 7 studies (6 studies used Nanostring nCounter^®^ and 1 study used QuantityGene Plex assay) that reported high and excellent correlations on different probes (ranging from r = 0.66 and also exceeding r > 0.9; R^2^ = 0.89–0.96; CCC = 0.98). RNA-seq was used in 15 studies that reported high correlations on different levels (ranging from r = 0.589 and exceeding r > 0.9; R^2^ > 0.8; CCC = 0.63–0.96).

**Table 1:** Characteristics of the studies that explored the performance of RNA extracted from matched FF-FFPE tissue samples derived from patients with breast cancer.

Publication	Journal	No. of samples	Technology/ Platform	Correlation, concordances
Bibikova et al.^36^ (2004)	The American Journal of Pathology	2	DASL	N/A
Loudig et al.^37^ (2007)	Nucleic Acids Research	1	Microarray based	$R^2=0.10$
Duenwald et al.^38^ (2009)	Journal of Translational Medicine	50	Microarray based	$r_{s}=0.88$ (good templates, p < 0.001), $r_{s}=0.81$ (poor templates, p < 0.001)
Waddell et al.^39^ (2010)	The Journal of Pathology	15	DASL	Concordant prediction of the same intrinsic subtype (11/15 intrinsic list, 12/15 modified intrinsic list)
Kibriya et al.^40^ (2010)	BMC Genomics	N/A	DASL	$r_p^2=0.75$ (1 representative sample) Low concordance
Mittempergher et al.^41^ (2011)	PLoS One	20	DASL	$r_p=0.65-0.89 \, (\pm 0.03)$ all probes $r_p=0.70-0.80 \, (\pm 0.05)$ most informative probes $R^2=0.94$ (60-gene index) High concordance
Morrogh et al.^42^ (2012)	The Journal of Surgical Research	16	DASL	$r_p=0.82$ (median) range: $0.63-0.92$ Good correlation
Meng et al.^43^ (2013)	PLoS One	2	RNA-seq	(250 miRNAs with no missing data) $r_p=0.90$ and $r_p=0.85$ High correlations of miRNAs
Li et al.^44^ (2012)	Breast Cancer Research and Treatment	2	RNA-seq	$r_p=0.9909$ (p < 0.001) (invasive micropapillary carcinoma) $r_p=0.9904$ (p < 0.001) (invasive ductal carcinoma of no special types) Good correlation of miRNA expression
Norton et al.^45^ (2013)	PLoS One	9	NanoString RNA-seq	NanoString, 226 genes: $r_p=0.874$, $r_{s}=0.954$ Excellent correlation between all pairs RNA-seq whole transcriptome: $r_p=0.783$, $r_{s}=0.953$ RNA-seq lincRNA: $r_p=0.988$, $r_s=0.861$ Excellent correlation High correlation of gene expression with both platforms
Sapino et al.^46^ (2014)	The Journal of molecular diagnostics	20 211	Microarray based	$r_p = 0.881$ (n=10 low-risk profiles; 95% CI 0.815-0.925) $r_p = 0.832$ (n = 10 high-risk profiles; 95% CI 0.743–0.893) Very strong concordance (70-gene profile) $r_p = 0.917$ (n = 211; 95% CI 0.893–0.936) High correlation (MammaPrint indices)
Nishio et al.^47^ (2014)	Clinical breast cancer	25	Microarray based	$r_p = 0.63$ High correlation
Andrade et al.^48^ (2015)	Molecular Oncology	5	NanoString	Concordant
Zhao et al.^49^ (2014)	BMC Genomics	11	Microarray based RNA-Seq	(RNA-seq platform; Representative tumour sample) $r_p = 0.924$ for DSN-seq $r_p = 0.896$ for Ribo-Zero-Seq High concordance in trascript quantification
Musella et al.^50^ (2015)	PLoS One	19	Microarray based	N/A
Beumer et al.^51^ (2016)	Breast Cancer Research and Treatment	552	Microarray based	$r_p=0.93$; 95% CI 0.92–0.94 Excellent correlation
Jovanović et al.^52^ (2017)	BMC Cancer	21	RNA-Seq	$r_s = 0.83 \pm 0.08$ (n=11 using MiSeq) $r_s = 0.88 \pm 0.04$ (n=10 using HiSeq) High similarity between gene expression profiles
Loudig et al.^53^ (2017)	International Journal of Molecular Sciences	44	RNA-seq	$r_p > 0.93$ (after batch correction)
Yamaguchi et al.^54^ (2018)	Oncotarget	5	NanoString	$r_p > 0.9$ Good correlations
Jose et al.^55^ (2018)	PLoS One	8	Microarray based	High concordance ( 50% gene modules)
Loudig et al.^56^ (2018)	Journal of Visualized Experiments	2	RNA-Seq	High correlation
Wrzeszczynski^57^$$^{*}$$ (2018)	The Journal of Molecular Diagnostics	3 (DNA) 3 (RNA)	RNA-seq (RNA) NGS (DNA)	Concordant
Li et al.^58^ (2018)	JCO Precision Oncology	9	RNA-Seq	$r_s > 0.85$ High concordance
Stewart et al.^59^ (2019)	Cancer Research	3	NanoString	$r_s^2 = 0.89 - 0.96; p < 0.0001$ High correlation
Marczyk et al.^60^ (2019)	BMC Cancer	12	RNA-seq	CCC = 0.63–0.66 (whole transcriptome) CCC = 0.91–0.96 (targeted 31 transcripts) Concordant
Turnbull et al.^61^ (2020)	BMC Bioinformatics	7	Microarray based NanoString RNA-Seq	Highly concordant (after batch correction)
Sun et al.^62^ (2020)	The Journal of Surgical Research	28	NanoString	$r_p = 0.66; p < 0.001$ Malignancy-Risk 117 gene-signature
Lau et al.^63^ (2020)	Clinical Chemistry	61	QuantiGene-Plex Assay	$R^2 = 0.96$ (for SET 2,3 index) CCC = 0.98 High concordance
Bergeron et al.^64^ (2022)	Journal of Molecular Medicine	8	RNA-Seq	N/A
Liu et al.^65^ (2022)	BMC Medical Genomics	7	RNA-Seq	N/A
Hilmi et al.^66^ (2022)	Current Issues in Molecular Biology	20	RNA-Seq	N/A
Marczyk et al.^67^ (2023)	Cancer Cytopathology	11	RNA-Seq	CCC = 0.627 $r_p = 0.831$

$R^2$ coefficient of determination, $r_{p}$ Pearson correlation coefficient, $r_{s}$ Spearman correlation coefficient, *CCC* Lin’s concordance correaltion coefficient.

*Study that tested the performance of both nucleic acids.

Among the nine studies that explored the performance of DNA extracted from matched FF-FFPE BC tissue material (Table 2), the degree of concordance was not reported in three studies. DNA sequencing with chain-terminating inhibitors (Sanger) was used in two studies, however the degree of concordance was not reported in either study. Next-Generation sequencing was used in five studies that reported varying correlations on different genome levels ranging from poor concordance in low-sequenced regions to high and excellent concordance in more reliable genome regions.

**Table 2:** Characteristics of the studies that explored the performance of DNA extracted from matched FF-FFPE samples derived from patients with breast cancer.

Publication	Journal	No. of samples	Technology/platform	Correlation, concordances
MacConaill et al.^68^ (2009)	PLoS One	20	Sanger	N/A
Schweiger et al.^69^ (2009)	PLoS One	1	NGS	89.8% common SNPs
Bourgon et al.^70^ (2014)	Clinical Cancer Research	4	NGS	Excellent concordance
Munchel et al.^71^ (2015)	Oncotarget	2	NGS	(Whole genome, whole exome and targeted exon sequencing) High concordance
Martelotto et al.^72^ (2017)	Nature Medicine	N/A	NGS	(Single-nuclei whole-genome copy number profiling; 4 matched cases, in total, after batch correction) High concordance
Wrzeszczynski et al.^57^$$^{*}$$ (2018)	The Journal of Molecular Diagnostics	3 (RNA) 3 (DNA)	RNA-seq (RNA) NGS (DNA)	Concordant
Robbe et al.^73^ (2018)	Genetics in Medicine	10	NGS	Poor concordance (in regions of low sequence complexity and reduced read mappability) High concordance (in reliable regions representing 69% of the genome)
Nachmanson et al.^74^ (2020)	BMC Medical Genomics	1	Sanger	N/A
Wei et al.^75^ (2021)	Gigascience	13	NGS	N/A

*Study that tested the performance of both nucleic acids.

### Analytical performance of different DNA/RNA extraction protocols in archival FFPE patient material

**Figure 2:**
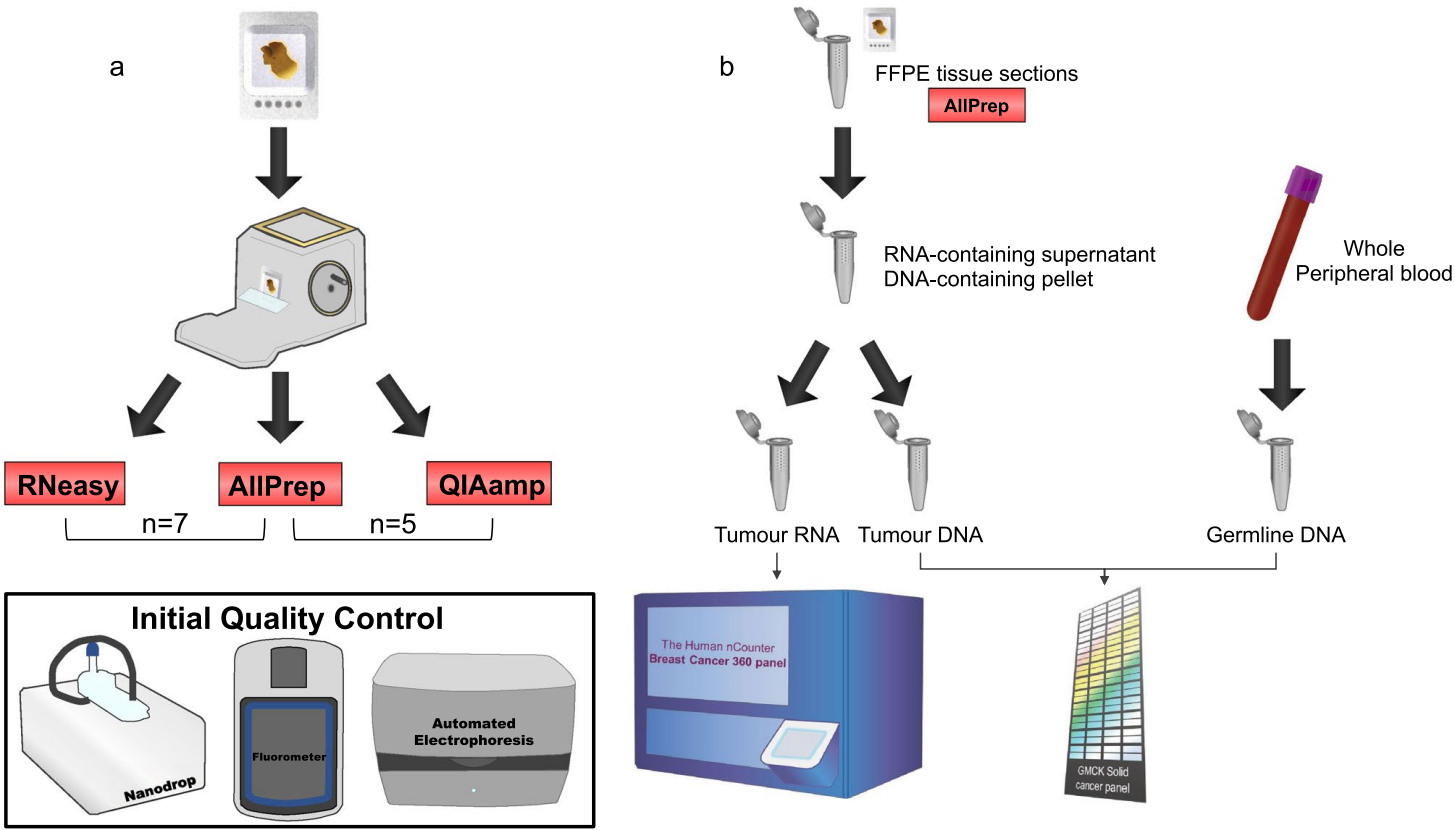
Experimental workflow including (**a**) the evaluation of three commercially available kits for the extraction of nucleic acids from FFPE BC tissue material sole DNA (QIAamp), sole RNA (RNeasy), and simultaneously DNA and RNA (AllPrep). The AllPrep and the RNeasy were used for nucleic acid extraction of seven (n = 7) archival FFPE BC tissue blocks, while the QIAamp was used only for DNA extraction from five (n = 5) of these seven FFPE blocks. The extracted nucleic acids underwent an Initial Quality Control evaluation using Nanodrop spectrophotometer, Qubit fluorometer and 2200 Tapestation automated electrophoresis system. (**b**) Tumour DNA extracted from thirty (n = 30) tumour-rich surgical FFPE BC tissue blocks derived from the SBG 2004-1 phase II clinical trial using the AllPrep, followed by targeted DNA GMCK panel, while matched germline DNA extracted from whole peripheral blood using FlexiGene DNA kit was used as control. Tumour RNA samples (n = 2) were analyzed using the Human nCounter Breast Cancer 360 gene panel and the nSolver Analysis Software version 4.0.

Based on the aforementioned results, we aimed to evaluate and compare mainly the nucleic acid yield using three different commonly used and commercially available kits for DNA only, RNA only and simultaneous DNA/RNA extraction from archival surgical FFPE tissue material derived from patients with early BC. The overall experimental workflow is depicted in Fig. 2 and the selection of the kits was based on the studies previously reporting high degree of concordance. RNA was extracted from 7 surgical FFPE tissue samples using consecutive sections for both RNeasy FFPE (RNeasy) and AllPrep DNA/RNA FFPE (AllPrep) kits. Quantification using the Nanodrop spectrophotometer revealed that the AllPrep extraction kit yielded slightly higher RNA compared to RNeasy (mean: 245.4 ng/$\upmu$L vs 209.8 ng/$\upmu$L, respectively) (paired t-test p = 0.553), but similar RNA yield based on Qubit fluorometer (mean: 175.4 ng/$\upmu$L vs 182.6 ng/$\upmu$L, respectively) (paired t-test p = 0.873). DNA was extracted from 5 surgical FFPE tissue samples using consecutive sections for both QIAamp DNA FFPE Tissue (QIAamp) and AllPrep extraction kits. Yield estimation based on Nanodrop spectrophotometer showed that the AllPrep extraction kit yielded similar DNA compared to QIAamp (mean: 130.6 ng/$\upmu$L vs 125.6 ng/$\upmu$L, respectively) (paired t-test p = 0.851). Similar yield between the AllPrep and QIAamp was also estimated based on Qubit fluorometer (mean: 61.8 ng/$\upmu$L vs 49.6 ng/$\upmu$L, respectively) (paired t-test p = 0.425). More information regarding the age, series of consecutive sections, material type and all initial QC metrics are available as Supplementary Material S1.

### Performance of the simultaneous DNA/RNA extraction protocol and targeted DNA sequencing in archival FFPE clinical samples

Considering the similar performance among the extraction kits, solely based on the initial QC metrics, we aimed to demonstrate the feasibility of downstream applications for the simultaneously extracted DNA/RNA using AllPrep, comparable to the findings of the systematic literature review. We thus used archival surgical FFPE tissue and matched whole peripheral blood from thirty patients previously enrolled in the SBG2004-1 study. After using AllPrep kit and two FFPE sections of 10 $\upmu$m in thickness as starting material for each of the samples, initial QC was performed, while the metric measurements are provided as Supplementary Material S1.

**Figure 3:**
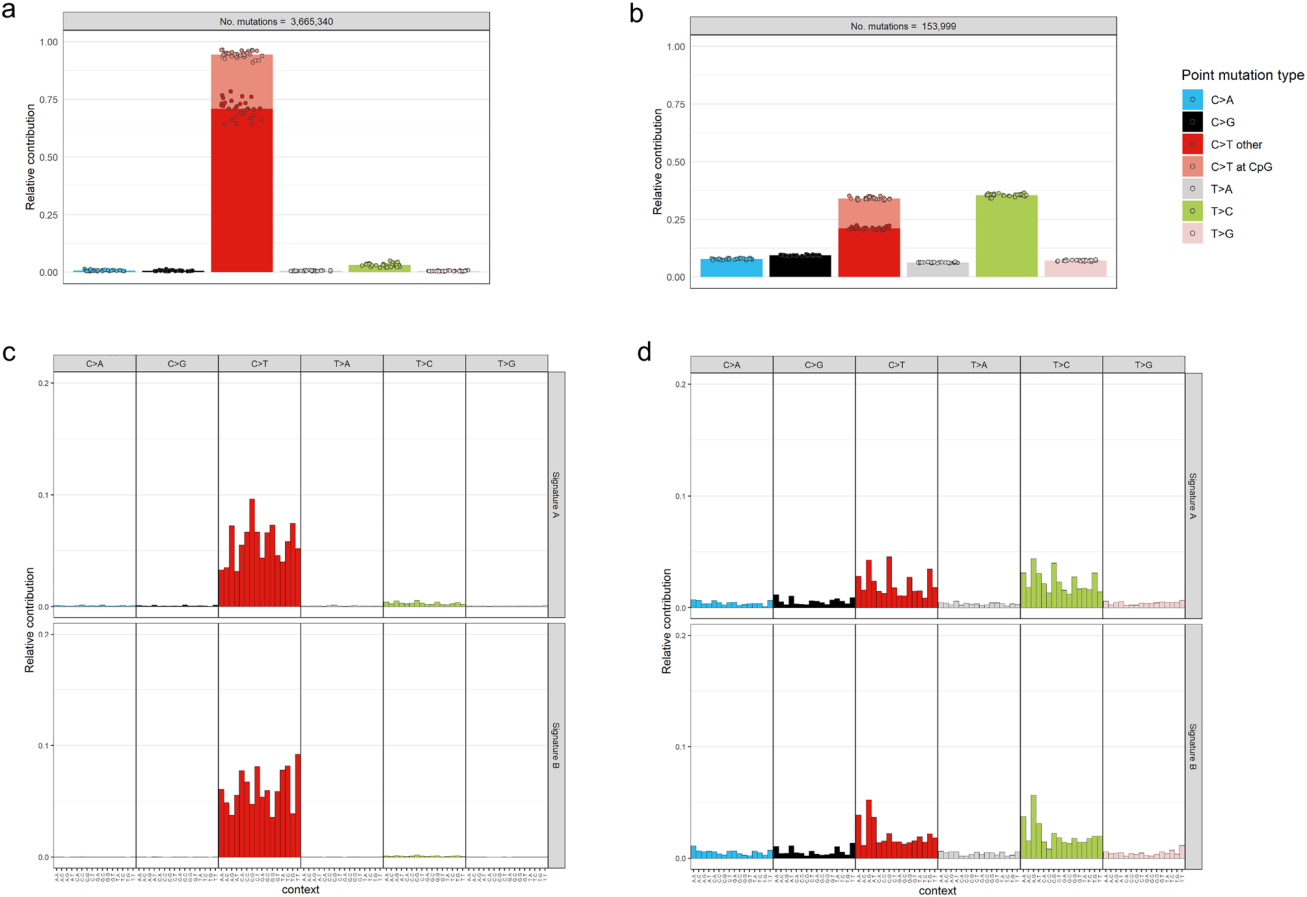
Substitution frequency barplots across samples show the relative contribution of the indicated mutation types to the point mutation spectrum for VarDict caller, (**a**) before and (**b**) after excluding variants below 5% variant allele frequency^32^. Before filtering, samples show high proportion of C>T substitutions. Bars depict the mean relative contribution of each mutation type over all the samples. The total number of somatic point mutations across samples, after tumour-normal paired analysis of 30 patients, is indicated for VarDict caller. Two de novo mutational signatures extracted based on non-negative matrix factorization (NMF) using VarDict caller, (**c**) before and (**d**) after excluding variants below 5% variant allele frequency. Substitution frequency barplots depict the mean relative contribution of each mutation type over all the samples, after tumour-normal paired analysis of 30 patients, is indicated for VarDict caller. Overall, the mutation pattern analysis show high proportion of C>T substitutions before filtering.

Tumour DNA and matched germline DNA samples derived from 30 patients with primary BC were sequenced using the GMCK Solid Cancer Panel. The variant caller algorithm VarDict was used for mutational pattern analysis and two de novo mutational signatures (Fig. 3). Before excluding variants below 5% variant allele frequency, VarDict detected 3,665,340 somatic SNVs in total, with very high proportion of C>T substitutions and very low proportion for all other point mutation across the samples (Fig. 3a). After excluding variants below 5% variant allele frequency, VarDict detected 153,999 somatic SNVs in total across the samples (Fig. 3b). The relative contribution of C>T substitutions was substantially lower after excluding variants below 5% variant allele frequency compared to prior filtering. The opposite effect was observed for all other point mutation types. The two de novo mutational signatures were also extracted based on non-negative matrix factorization for the VarDict caller, before (Fig. 3c) and after excluding variants below 5% variant allele frequency (Fig. 3d). Based on the mutation pattern analysis, before filtering samples had the highest proportion of C>T substitutions compared to post-filtering.

### Performance of the simultaneous DNA/RNA extraction protocol and gene expression profiling in archival FFPE clinical samples

Gene expression analysis of the RNA extracted from two patients enrolled in the SBG2004-1 clinical trial was performed using nCounter^®^ Breast Cancer 360^™^ gene panel. The RNA yield was adequate for both samples, while 300 ng input was required for running the assay. Standardized quality assurance metrics calculation and data pre-processing revealed no QC flags or other performance issues. Moreover, all gene transcript probes exhibit the background threshold which was determined by the negative control probes included in the BC360 panel. The gene expression distribution of the top 20 endogenous genes and negative controls is illustrated in box plots (provided as Supplementary Material S1), while all QC metrics concerning the nCounter BC360 panel are summarized in a table (provided as Supplementary Material S1). All generated raw and pre-processed gene expression data for the 776 gene targets are listed as Supplementary Material S1.

## Discussion

High-throughput downstream application technologies represent powerful tools for understanding cancer biology and developing clinically valuable biomarkers and targeted therapies. Given that archival FFPE material is often the only available tissue source for translational research studies, its challenging nature can limit the performance of such genomic and transcriptomic methods. The present study focusing on archival FFPE BC tissue, represents a multi-level approach including: (i) a systematic literature search of the studies comparing FF and FFPE-matched BC tissue patient material; (ii) experimental comparison and evaluation of three commercially available DNA/RNA extraction kits and (iii) feasibility study of the simultaneous DNA/RNA extraction protocol using archival tissue from patients enrolled in a randomized trial for targeted DNA-sequencing and gene expression analysis.

A high degree of concordance between FF and FFPE-matched tissue samples derived from patients with BC was observed for both RNA and DNA analyses according to the published literature. The highest degree of concordance among different platforms for gene expression analysis was reported with nCounter, a hybridization-based platform (Nanostring), showing credibility for low yielded RNA samples^45,48,54,59,61,62^. For genomic analysis, although a few studies reported comparison metrics on the performance of DNA-seq between FF and FFPE-matched material, the degree of concordance as reported in individual studies remained high. Of note, this concordance is constantly improving over the years, indicating the evolution of already available and dynamic development of new powerful sequencing technologies and analysis tools.

In our study, the simultaneous DNA/RNA extraction method (AllPrep) compared to RNA-only (RNeasy) and DNA-only (QIAamp) extraction methods performed similarly in all three initial QC metrics, including purity assessment, quantification, and integrity estimation of the nucleic acids. The initial QC metrics of the nucleic acids extracted from FFPE material are pertinent. Despite the purity ratios, $A_{260}$/$A_{280}$ and $A_{260}$/$A_{230}$, and the relatively low integrity of the nucleic acids, a more important factor when it comes to FFPE sample selection for certain technique remains the yield assessment so as to calculate the input amount. For all three silica-based extraction methods, DNA yield based on Nanodrop quantification was up to 3-fold higher compared to Qubit, while a lower discrepancy was noticed for RNA yield. The fluorescence-based nucleic acid quantification method (i.e., Qubit fluorometer) seems to be more sensitive compared to spectrophotometer-based methods, possibly due to the selective binding mode of the fluorescent dyes on different nucleic acids. Spectrophotometer’s nucleic acid quantification algorithms are more prone to overestimate the nucleic acid yield due to their disadvantage in distinguishing ribonucleotide and deoxyribonucleotide formations. In addition to the DIN and RIN$^{e}$ values, the DV$_{200}$ value has been proposed as an additional quality metric for the estimation of RNA integrity^76^. A weak correlation between different RNA integrity metrics is noticed in low-yield FFPE samples compared to FF^77^, calling both RIN$^{e}$ and DV$_{200}$ metrics in question regarding the qualification for downstream applications^78^.

In order to demonstrate the feasibility of the simultaneous DNA/RNA extraction method (AllPrep) in the context of an early breast cancer clinical trial, targeted DNA sequencing was performed using a hybrid capture-based panel for genomic DNA extracted from tumour-rich surgical FFPE blocks and for matched germline DNA extracted from whole peripheral blood. Although the libraries were constructed successfully, the bioinformatic analysis of the NGS data had to be optimised in order to overcome potential artefacts due to the challenging nature of the FFPE samples. The high C:G>T:A transitions appeared to be an artefact of FFPE samples due to cytosine deamination^30^. To better interpret the sequencing data, mutation pattern analysis was performed using the VarDict variant caller algorithm, before and after excluding variants below 5% variant allele frequency. The latter is a previously suggested post-filtering strategy^32^ to remove the high proportion of C>T/G>A. We have achieved similar relative contributions of the indicated mutation types to the point mutation spectrum, as previously reported^32^. Several pre-sequencing method approaches have been proposed to improve the accuracy of DNA sequencing data, such as the pretreatment with several DNA glycosylases^79–83^.

Tissue morphology is better preserved in FFPE biospecimens, improving pathology evaluation compared to FF. Additionally, long-term storage in non-sterile conditions requires less maintenance and fewer resources compared to FF. On the other hand, nucleic acids extracted from FFPE are often degraded and prone to artefacts compared to FF^2,3^. Therefore, the collection of both FF and FFPE tissue material is preferable, in order to minimize pre-analytical variability of prospectively collected tissues^84^.

One of the limitations of this study is publication bias which may have led to overestimation of the correlation between FF and FFPE samples. Another limitation of the systematic review is that it only included studies that reported on specific platforms and methods. Therefore, nucleic acid analysis technique platforms like other PCR-based methods (e.g., qPCR, RT-qPCR, hydrolysis of pre-existing sequences TaqMan^®^), DNA methylation analysis methods (e.g., quantitative bisulfite pyrosequencing), chromatin immunoprecipitation sequencing (i.e., ChIP-Seq) were not included in our study. Moreover, the studies reported performance comparisons between FF and FFPE-matched nucleic acid material, often descriptively and at different levels, making it difficult to further analyze and draw certain conclusions. For the pre-analytical performance part, a limitation is that the compared nucleic acid extraction kits are of the same silica gel membrane technology, while no other extraction kits based on paramagnetic particles or glass fibers were tested^78,85^. Further, due to the low sample size, no significant differences in QC metrics were obtained when comparing the AllPrep with the other two kits. The feasibility study workflow, using the simultaneous DNA/RNA extraction method on archival surgical FFPE material was demonstrated by the performance of the extracted DNA using the targeted DNA GMCK panel for thirty patients, while its performance for the extracted RNA was demonstrated using nCounter BC360 panel only for two patients.

## Conclusion

The present study highlights the importance of optimising the pre-analytical variables and the QC metrics to evaluate better the yield, purity and integrity of the nucleic acids extracted from FFPE samples. The initial QC would be of particular interest when applying costly high-throughput platforms on large-scale clinical trial material. Overall, we demonstrated the feasibility of simultaneous DNA and RNA extraction method from FFPE material using downstream applications, which are also highlighted in the systematic literature search part with high to excellent concordance. We also address the need in bioinformatics to exclude variants below 5% variant allele frequency to overcome FFPE-induced artefacts. This feasibility study-workflow, including targeted DNA NGS and gene expression analysis, serves as a pilot study for larger trials. In conclusion, our findings might provide input to translational studies where FFPE material is the only available patient tumour tissue resource.

### Supplementary Information

Supplementary Information 1.

Supplementary Information 2.

Supplementary Information 3.

Supplementary Information 4.

Supplementary Information 5.

## Data Availability

The raw and pre-processed gene expression data for the 776 gene targets generated in this study are included in the Supplementary Material. The raw DNA sequencing data files that support the findings of this study can be obtained from the corresponding author (D.S.) upon reasonable request, provided that the intended use aligns with the ethics approval and the informed consent signed by the trial participants.
