# Supplementary material for: Systematic review and feasibility study on pre-analytical factors and genomic analyses on archival formalin-fixed paraffin-embedded breast cancer tissue
Source: Sci Rep. 2024 Aug 6;14:18275. doi: 10.1038/s41598-024-69285-8 (PMC11303707; doi:10.1038/s41598-024-69285-8)
Supplement: Supplementary file 1 — Supplementary Information 1. [file 41598_2024_69285_MOESM1_ESM.pdf]

Karolinska Institutet

Documentation of search strategies
University Library search consultation group

Date: February 2023 – updated search

Topic/research question: **To evaluate the degree of concordance and/or the success rate between Fresh-Frozen (FF) and FFPE breast cancer biospecimens for genomic and gene expression applications.**

Name of researcher(s): Dimitrios Salgkamis & Ioannis Zerdes, Oncology - Pathology

Librarian(s): GunBrit Knutssön & Narcisa Hannerz (GB preformed original search with peer review from N. N preformed update of search)

Databases:

1. Medline(OVID)
2. Embase.com
3. Web of Science(Clarivate)
4. PubMed Central

Total number of hits:

Medline(OVID), Embase,com & Web of Science

- Before deduplication: 3,692 (415 of them new references from update)
- After deduplication: 2,204 (272 of them new references from update)

---

1. Medline

Interface: Ovid<br><br>Date of Search: 2 Feb 2023<br><br>Number of hits: 885<br><br>Comment: In Ovid, two or more words are automatically searched as phrases; i.e. no quotation marks are needed	Field labels<br><br>• exp/ = exploded MeSH term<br>• / = non exploded MeSH term<br>• .ti,ab,kf. = title, abstract and author keywords<br>• adjx = adjacent within x words, regardless of order<br>• * = truncation of word for alternate endings

1. Paraffin Embedding/
2. Formaldehyde/
3. ((formaldehyde or formalin or paraffin) adj3 (embed* or fix* or preserv*)).ti,ab,kf.
4. FFPE.ti,ab,kf.
5. Frozen Sections/
6. ((freeze or frozen) adj3 (fresh or section* or snap*)).ti,ab,kf.
7. or/1-6

8. exp Breast Neoplasms/
9. ((breast or mammary) adj3 (carcinom* or cancer* or neoplasm* or tumor* or tumour*)).ti,ab,kf.
10. or/8-9

11. DNA Copy Number Variations/
12. exp High-Throughput Nucleotide Sequencing/
13. Multiplex Polymerase Chain Reaction/
14. Nanopore Sequencing/
15. Oligonucleotide Array Sequence Analysis/
16. Polymorphism, Single Nucleotide/
17. Real-Time Polymerase Chain Reaction/
18. Reverse Transcriptase Polymerase Chain Reaction/
19. Whole-Genome Sequencing/
20. ((chain reaction or PCR) adj3 (kinetic or multiple or real-time or transcriptase or triplex)).ti,ab,kf.
21. ((DNA or cDNA) adj3 (array* or copy or microarray* or microchip* or polymorphism)).ti,ab,kf.
22. (oligonucleotide array* or probe amplification or RNA-seq or single nucleotide).ti,ab,kf.
23. (Clariom S assay* or TempO-seq or CUTSeq or Illumina Beadchip or Ion AmpliSeq or QuantSeq or nanostring or Qiagen or QiaSeq).ti,ab,kf.
24. (sequenc* adj3 (deep or genome or high-throughput or illumina or nanopore or ion proton or ion torrent or massively-parallel or next-generation or shotgun)).ti,ab,kf.
25. or/11-24

26. 7 and 10 and 25

27. 26 not (animals not humans).sh.
28. limit 27 to (clinical conference or congress or consensus development conference or consensus development conference, nih or editorial or letter or news)
29. 27 not 28
30. limit 29 to english language

Karolinska Institutet

2. Embase

Interface: embase.com<br><br>Date of Search: 2 Feb 2023<br><br>Number of hits: 1,128<br><br>Comment: Emtree is the controlled vocabulary in Embase	Field labels<br><br>• /exp = exploded Emtree term<br>• /de = non exploded Emtree term<br>• ti,ab = title and abstract<br>• NEAR/x = adjacent within x words, regardless of order<br>• * = truncation of word for alternate endings

('paraffin embedding'/de OR 'formaldehyde'/de OR (((formaldehyde OR formalin OR paraffin) NEAR/3 (embed* OR fix* OR preserv*)):ti,ab,kw) OR ffpe:ti,ab,kw OR 'frozen section'/de OR (((freeze OR frozen) NEAR/3 (fresh OR section* OR snap*)):ti,ab,kw))

AND

('breast tumor'/exp OR (((breast OR mammary) NEAR/3 (carcinom* OR cancer* OR neoplasm* OR tumor* OR tumour*)):ti,ab,kw))

AND

('copy number variation'/de OR 'high throughput sequencing'/exp OR 'multiplex polymerase chain reaction'/exp OR 'dna microarray'/de OR 'single nucleotide polymorphism'/de OR 'real time polymerase chain reaction'/de OR 'reverse transcription polymerase chain reaction'/de OR 'whole genome sequencing'/de OR ((('chain reaction' OR pcr) NEAR/3 (kinetic OR multiple OR 'real time' OR transcriptase OR triplex)):ti,ab,kw) OR (((dna OR cdna) NEAR/3 (array* OR copy OR microarray* OR microchip* OR polymorphism)):ti,ab,kw) OR 'oligonucleotide array*':ti,ab,kw OR 'probe amplification':ti,ab,kw OR 'rna-seq':ti,ab,kw OR 'single nucleotide':ti,ab,kw OR 'clariom s assay*':ti,ab,kw OR 'tempo-seq':ti,ab,kw OR cutseq:ti,ab,kw OR 'illumina beadchip':ti,ab,kw OR 'ion ampliseq':ti,ab,kw OR 'quantseq':ti,ab,kw OR nanostring:ti,ab,kw OR qiagen:ti,ab,kw OR qiaseq:ti,ab,kw OR ((sequenc* NEAR/3 (deep OR genome OR 'high-throughput' OR illumina OR nanopore OR 'ion proton' OR 'ion torrent' OR 'massively-parallel' OR 'next-generation' OR shotgun)):ti,ab,kw))

NOT ([animals]/lim NOT [humans]/lim)

AND ([article]/lim OR [article in press]/lim OR [erratum]/lim OR [review]/lim) AND [english]/lim

3. Web of Science Core Collection

Interface: Clarivate Analytics<br><br>Date of Search: 2 feb 2023<br><br>Number of hits: 935	Field labels<br><br>• TS/Topic = title, abstract, author keywords and Keywords Plus<br>• NEAR/x = adjacent within x words, regardless of order<br>• * = truncation of word for alternate endings

#1 TS=(((formaldehyde or formalin or paraffin) NEAR/3 (embed* or fix* or preserv*))) OR TS=("FFPE") OR TS=((freeze or frozen) NEAR/3 (fresh or section* or snap*))

#2 TS=(((breast or mammary) NEAR/3 (carcinom* or cancer* or neoplasm* or tumor* or tumour*)))

#3 TS=(((”chain reaction” or PCR) NEAR/3 (kinetic or multiple or real-time or transcriptase or triplex))) OR TS=(((DNA or cDNA) NEAR/3 (array* or copy or microarray* or microchip* or polymorphism))) OR TS=((”oligonucleotide array*” or ”probe amplification” or ”RNA-seq” or ”single nucleotide”)) OR TS=((”Clariom S assay*” or ”TempO-seq” or CUTSeq or ”Illumina Beadchip” or ”Ion AmpliSeq” or ”QuantSeq” or nanostring or Qiagen or QiaSeq)) OR TS=((sequenc* NEAR/3 (deep or genome or ”high-throughput ”or illumina or nanopore or ”ion proton” or ”ion torrent” or ”massively-parallel” or ”next-generation” or shotgun))

#4 #1 AND #2 AND #3 **AND and Article or Early Access or Review Article (Document Types) and English (Languages)**

4. PubMed Central

Date of Search: 2 Feb 2023<br><br>Number of hits: 744<br><br>Comment:	Field labels<br><br>• * = truncation of word for alternate endings<br>• [Body – All Words] = searching full-text<br>• [title] = searching title<br>• [abstract] = searching abstract<br>• [MeSH term] = searching Mesh terms<br>•

((((Paraffin Embedding[mesh terms] OR paraffin embed*[abstract] OR formalin-fixed paraffin-embedd*[abstract] OR FFPE[abstract] OR Frozen Sections[mesh terms] or frozen section*[abstract] OR fresh-frozen[abstract] OR fresh freeze[abstract] OR snap-frozen[abstract])))

AND

(((breast carcinom*[body - all words] OR breast cancer*[body - all words] OR breast neoplasm*[body - all words] or breast tumor*[body - all words] OR breast tumour*[body - all words]) NOT ((breast carcinom*[title] OR breast cancer*[title] OR breast neoplasm*[title] or breast tumor*[title] OR breast tumour*[title])))

AND

((DNA Copy Number Variations[mesh term] OR DNA Copy Number[abstract] High-Throughput Nucleotide Sequencing[mesh term] OR High-Throughput[abstract] OR Deep Sequencing[abstract] OR Illumina Sequencing[abstract] OR Ion Proton Sequencing[abstract] OR Ion Torrent Sequencing[abstract] OR Massively-Parallel Sequencing[abstract] OR Next-Generation Sequencing[abstract] OR Multiplex Polymerase Chain Reaction[mesh term] OR Multiplex PCR[abstract] OR Triplex PCR[abstract] OR Triplex Polymerase Chain Reaction[abstract] OR Nanopore Sequencing[mesh term] OR Oligonucleotide Array Sequence Analysis[mesh term] OR DNA Array*[abstract] OR DNA Chip[abstract] OR DNA Microarray*[abstract] OR DNA Microchip*[abstract] OR Gene Chip*[abstract] OR Oligonucleotide Array*[abstract] OR Oligonucleotide Microarray*[abstract] OR cDNA Array*[abstract] OR cDNA Microarray*[abstract] OR Polymorphism, Single Nucleotide[mesh term] OR Single Nucleotide Polymorphism[abstract] OR Real-Time Polymerase Chain Reaction [mesh term] OR Kinetic Polymerase Chain Reaction[abstract] OR Real-Time PCR[abstract] OR Reverse Transcriptase Polymerase Chain Reaction[mesh term] OR Reverse Transcriptase PCR[abstract] OR RNA-Seq[mesh term] or RNA-Seq[abstract] OR Whole Genome Sequencing[mesh term] OR Whole- genome sequencing[abstract] or Complete Genome Sequencing[abstract]) OR (Clariom S[abstract] OR TempO-seq[abstract] OR CUTSeq[abstract] OR Illumina Beadchip[abstract] OR Ion AmpliSeq[abstract] OR QuantSeq[abstract] OR nanostring[abstract] OR Qiagen[abstract] OR QiaSeq[abstract]))

NOT ((animals NOT humans))
