# Supplementary material for: Systematic review and feasibility study on pre-analytical factors and genomic analyses on archival formalin-fixed paraffin-embedded breast cancer tissue
Source: Sci Rep. 2024 Aug 6;14:18275. doi: 10.1038/s41598-024-69285-8 (PMC11303707; doi:10.1038/s41598-024-69285-8)
Supplement: Supplementary file 2 — Supplementary Information 2. [file 41598_2024_69285_MOESM2_ESM.docx]

GMCK Solid cancer panel description

# Applications

The 370-gene panel has been designed to enable detection of clinically relevant single-nucleotide variants (SNVs) and small insertion/deletion variants (INDELs), copy-number aberrations (CNAs), fusion events (fusions), microsatellite instability (MSI) and to estimate the tumour mutational burden (TMB) in a single assay. The panel also contains selected hotspot variants in genes where there is strong evidence of pharmacogenetic relevance.

# Design principles

The panel has been designed through an extensive process assessing the scientific and medical evidence supporting the inclusion of each gene in the panel. The design process considered extent of mutations, clinical actionability, mutation frequency in TCGA, and also knowledge of pathogenic germline variants. Genes were categorised as relevant for SNV/INDEL, CNA or fusion detection, then scored based on their frequency in the various datasets, and finally manually assessed for inclusion of the entire coding region, or of only selected hotspot loci. The ultimate aim of the panel was to represent the cancer genome in a mini-genome format. The panel has been designed using reference genome hg19.

# Content description

The panel, summarised in Table 1, includes 198 genes with entire protein-coding sequence, 132 genes with known hotspot regions, and nine genes (e.g. BRCA1, BRCA2, CDK12, TP53) with the entire gene body (coding + introns). Furthermore, for 19 genes introns are selected to enable detection of fusion events on the DNA level. The TERT promoter is also included in the design. A selection of 2800 SNPs were included to target the genome-wide backbone for CNA detection. The design also includes 86 genes with additional baits to increase resolution. MSI is detected using a combination of 63 genome-wide microsatellite loci and additional microsatellites located in the introns of the genes selected for this panel. TMB detection is facilitated by the design size of the panel, 2.4 Mb. Pharmacogenetic variants included are in nine genes (e.g. DPYD, COMT, CYP2D6, HSD3B1).

*Table 1*

| Design summary | | |
| --- | --- | --- |
|  | All coding exons | 198 genes |
|  | Hotspots | 132 genes |
| Pharmacogenetic variants | |  |
|  | SNPs | 9 genes |
| Copy-number alterations | |  |
|  | Tiled SNP for genome-wide CNV | 2814 SNPs |
|  | Directed analysis to increase sensitivity | 86 genes |
| Structural variation | |  |
|  | Gene fusions by intronic sequencing | 19 genes |
|  | Gene-body sequencing (e.g. BRCA1/2) | 9 genes |
| Microsatellite instability & Hypermutation | |  |
|  | Microsatellites | 63 in total |
|  | Hypermutation, entire design footprint | yes |
|  | Associated genes | 7 |
| Total size (Mb) | |  |
|  |  | 2.4 |

# Intended usage

The GMCK solid tumour panel is intended for genetic screening of solid tumours using either tumour-only or tumour-normal strategy. Both fresh-frozen (FF) and formalin-fixed paraffin embedded (FFPE) material can be used. The quality of results for FFPE materials may vary based on how the sample has been treated and the age of the specimen. This panel is not suitable for use with circulating cell-free DNA due to its design size.

The recommended minimum input quantity of material is 50 ng (up 250 ng will be used if available), although input quantities down to 10 ng may yield relevant results. Archived materials will/may result in reduced performance and it should be expected as such.

The recommended sequencing depth is 1000x average coverage (deduplicated) across the targeted regions. Due to variability of sequencing between the different samples in a project, this should ensure that the majority of the samples have been covered at 500x or more, which should allow detection of variants down to 2% allele frequency (10 observations in a total depth of 500x). Coverage for archived materials might not achieve these coverage levels, and the results will/may vary due to age and quality of specimen.

# Appendix 1 - Full panel content description

| **Full coding** | ACVR1B | BARD1 | CHEK1 | ERCC4 | H3F3C | LATS2 | NF1 | POLD1 | RECQL4 | STK11 |
| --- | --- | --- | --- | --- | --- | --- | --- | --- | --- | --- |
|  | ALB | BLM | CHEK2 | ESR1 | HGF | LZTR1 | NF2 | POLQ | RET | TAF1 |
|  | ALK | BMPR1A | CIC | EZH2 | HIST1H1E | MAP2K1 | NKX3-1 | POT1 | RNF43 | TBL1XR1 |
|  | AMER1 | BRCA1 | COL5A1 | FANCA | HLA-A | MAP2K4 | NOTCH1 | PPM1D | RPL5 | TBX3 |
|  | APC | BRCA2 | CREBBP | FANCC | HLA-B | MAP3K1 | NOTCH2 | PRKAR1A | SDHA | TCF12 |
|  | AR | BRIP1 | CSDE1 | FANCM | HLA-C | MED12 | NTRK1 | PRSS1 | SDHB | TCF7L2 |
|  | ARAF | BTG2 | CTCF | FAT1 | IL6ST | MEN1 | NTRK3 | PSIP1 | SDHC | TGFBR2 |
|  | ARHGAP35 | CASP8 | CTNNB1 | FBXW7 | IRF2 | MET | PALB2 | PTCH1 | SDHD | TLR4 |
|  | ARID1A | CD274 | CYLD | FGFR1 | JAK1 | MGA | PAX5 | PTEN | SETD2 | TP53 |
|  | ARID2 | CD70 | CYSLTR2 | FGFR2 | JAK2 | MITF | PBRM1 | RAD21 | SMAD2 | TRAF3 |
|  | ARID5B | CDC27 | DDX3X | FGFR3 | KDM5C | MLH1 | PDCD1 | RAD50 | SMAD4 | TSC1 |
|  | ASXL1 | CDH1 | DICER1 | FH | KDM6A | MLH3 | PDGFRA | RAD51 | SMARCA1 | TSC2 |
|  | ASXL2 | CDK12 | EGFR | FOXA1 | KEAP1 | MRE11 | PHOX2B | RAD51B | SMARCA4 | USP9X |
|  | ATM | CDKN1A | ELF3 | FUBP1 | KEL | MSH2 | PIK3CA | RAD51C | SMARCB1 | VHL |
|  | ATR | CDKN1B | EP300 | GABRA6 | KIT | MSH3 | PIK3CG | RAD51D | SMC1A | WT1 |
|  | ATRX | CDKN2A | EPCAM | GATA3 | KMT2A | MSH6 | PIK3R1 | RAD52 | SMC3 | ZFHX3 |
|  | AXIN1 | CDKN2C | EPHA3 | GNA13 | KMT2B | MTOR | PLCB4 | RASA1 | SOX9 | ZMYM2 |
|  | AXIN2 | CHD1 | ERBB2 | GNAS | KMT2C | MUTYH | PLCG1 | RB1 | SPEN | ZMYM3 |
|  | B2M | CHD3 | ERCC2 | GPS2 | KMT2D | NBN | PMS1 | RBM10 | SPTA1 |  |
|  | BAP1 | CHD4 | ERCC3 | GREM1 | LATS1 | NCOR1 | PMS2 | RECQL | STAG2 |  |
|  |  |  |  |  |  |  |  |  |  |  |
| **Hotspots** | ACVR1 | CTLA4 | FOXO1 | IRS2 | NUP93 | RAC1 | SPRED1 |  | **Pharmaco-**  **genetic** | COMT |
|  | ACVR2A | CUL3 | FOXP1 | JUN | PAK7 | RAF1 | STAT3 |  |  | CYP2D6 |
|  | AKT1 | DAXX | GATA2 | KDR | PARP1 | RHEB | STK19 |  |  | DPYD |
|  | AKT2 | DIS3 | GLI1 | KLF4 | PDPK1 | RHOA | SUZ12 |  |  | FGFR4 |
|  | AKT3 | DNAJB1 | GNA11 | KNSTRN | PGR | RICTOR | TCEB1/ELOC |  |  | HOXB13 |
|  | ANKRD11 | DNMT1 | GNAQ | KRAS | PIK3CB | RIT1 | TCF3 |  |  | HSD3B1 |
|  | ARID1B | DNMT3B | GSK3B | LYN | PIK3CD | RPL22 | TET1 |  |  | NQO1 |
|  | AXL | EIF1AX | GTF2I | MAP2K2 | PIK3R2 | RPS6KA4 | TGFBR1 |  |  | TPMT |
|  | BCL10 | EIF4A2 | H3F3A | MAP3K13 | PIM1 | RPTOR | TNFRSF14 |  |  | UGT1A1 |
|  | BCL2L11 | EPAS1 | HIST1H1C | MAPK1 | PLK2 | RRAS2 | TP63 |  |  |  |
|  | BCL6 | EPHA7 | HIST1H3B | MAX | POLE | RXRA | TRAF7 |  |  |  |
|  | BCOR | EPHB1 | HIST1H3C | MDC1 | PPP2R1A | SDHAF2 | U2AF1 |  |  |  |
|  | BRAF | ERBB3 | HIST1H3H | MEF2B | PPP4R2 | SESN2 |  |  |  |  |
|  | BRD4 | ERBB4 | HNF1A | MST1 | PPP6C | SMAD3 |  |  |  |  |
|  | CBL | ERG | HRAS | MYC | PRDM14 | SMARCD1 |  |  |  |  |
|  | CCND1 | ERRFI1 | IDH1 | MYCN | PREX2 | SMO |  |  |  |  |
|  | CCND3 | ETV6 | IDH2 | MYOD1 | PRKCI | SOCS1 |  |  |  |  |
|  | CDK4 | FAM58A | IGF1R | NCOA3 | PTPRD | SOS1 |  |  |  |  |
|  | CDK6 | FGFR4 | INPPL1 | NFE2L2 | PTPRS | SOX17 |  |  |  |  |
|  | CRLF2 | FOXL2 | IRF4 | NRAS | PTPRT | SPOP |  |  |  |  |
|  |  |  |  |  |  |  |  |  |  |  |
| **Fusions** | ALK | **Gene body** | AR |  | **CNA** | AKT2 | CHD1 | KMT2C | NTRK1 | TP53 |
|  | BRAF |  | ATM |  |  | ALK | CHEK2 | KRAS | PARK2 | TSC1 |
|  | EGFR |  | BRCA1 |  |  | AMER1 | CSMD1 | LRP1B | PAX5 | TSC2 |
|  | ERG |  | BRCA2 |  |  | APC | DROSHA | MAP2K4 | PBRM1 | VHL |
|  | ETV6 |  | CD274 |  |  | APOBEC3B | EGFR | MCL1 | PDE4D | WT1 |
|  | EWSR1 |  | CDK12 |  |  | AR | ERBB2 | MDM2 | PDGFRA | WWOX |
|  | FGFR1 |  | PTEN |  |  | AR_ENHANCER | FANCA | MDM4 | PIK3CA | ZBTB16 |
|  | FGFR2 |  | RB1 |  |  | ARID1A | FANCC | MEN1 | PIK3R1 |  |
|  | FGFR3 |  | TP53 |  |  | ATM | FANCD2 | MET | PPM1D |  |
|  | GPR126 |  |  |  |  | AXIN1 | FBXW7 | MITF | PTEN |  |
|  | KIT |  |  |  |  | BRCA1 | FGFR1 | MLH1 | RAF1 |  |
|  | NTRK1 |  |  |  |  | BRCA2 | FLT3 | MSH2 | RB1 |  |
|  | NTRK3 |  |  |  |  | CCND1 | FLT4 | MYC | SKP2 |  |
|  | PDGFRA |  |  |  |  | CCNE1 | FOXA1 | MYCL | SMAD4 |  |
|  | PDGFRB |  |  |  |  | CD274 | GPC3 | MYCN | SMARCB1 |  |
|  | RET |  |  |  |  | CDK12 | IGF1R | NCOA3 | SOX2 |  |
|  | ROS1 |  |  |  |  | CDK4 | INTS4 | NF1 | STK11 |  |
|  | TERT |  |  |  |  | CDKN2A | JUN | NF2 | SUFU |  |
|  | TMPRSS2 |  |  |  |  | CDKN2B | KDM6A | NKX2 | T_E_DEL |  |
|  |  |  |  |  |  | CDKN2C | KIT | NKX3 | TERT |  |
