# Supplementary material for: Systematic review and feasibility study on pre-analytical factors and genomic analyses on archival formalin-fixed paraffin-embedded breast cancer tissue
Source: Sci Rep. 2024 Aug 6;14:18275. doi: 10.1038/s41598-024-69285-8 (PMC11303707; doi:10.1038/s41598-024-69285-8)
Supplement: Supplementary file 4 — Supplementary Information 4. [file 41598_2024_69285_MOESM4_ESM.docx]

| Supplementary Table 1. showing QC metrics of the nCounter BC360 panel. All probes included in the BC360 panel exhibit the background threshold, in both samples, while no QC flags were issued. | | |
| --- | --- | --- |
|  | Case 1 | Case 2 |
| **% Probes Above Threshold** | 100 | 100 |
| **QC Flag** | No | No |
| **mRNA Positive Normalization Flag** | No | No |
| **mRNA Content Normalization Flag** | No | No |
| **mRNA Positive Normalization Factor** | 0.98 | 1.03 |
| **mRNA Content Normalization Factor** | 0.79 | 1.35 |
|  | | |
