# Supplementary material for: Systematic review and feasibility study on pre-analytical factors and genomic analyses on archival formalin-fixed paraffin-embedded breast cancer tissue
Source: Sci Rep. 2024 Aug 6;14:18275. doi: 10.1038/s41598-024-69285-8 (PMC11303707; doi:10.1038/s41598-024-69285-8)
Supplement: Supplementary file 5 — Supplementary Information 5. [file 41598_2024_69285_MOESM5_ESM.pdf]

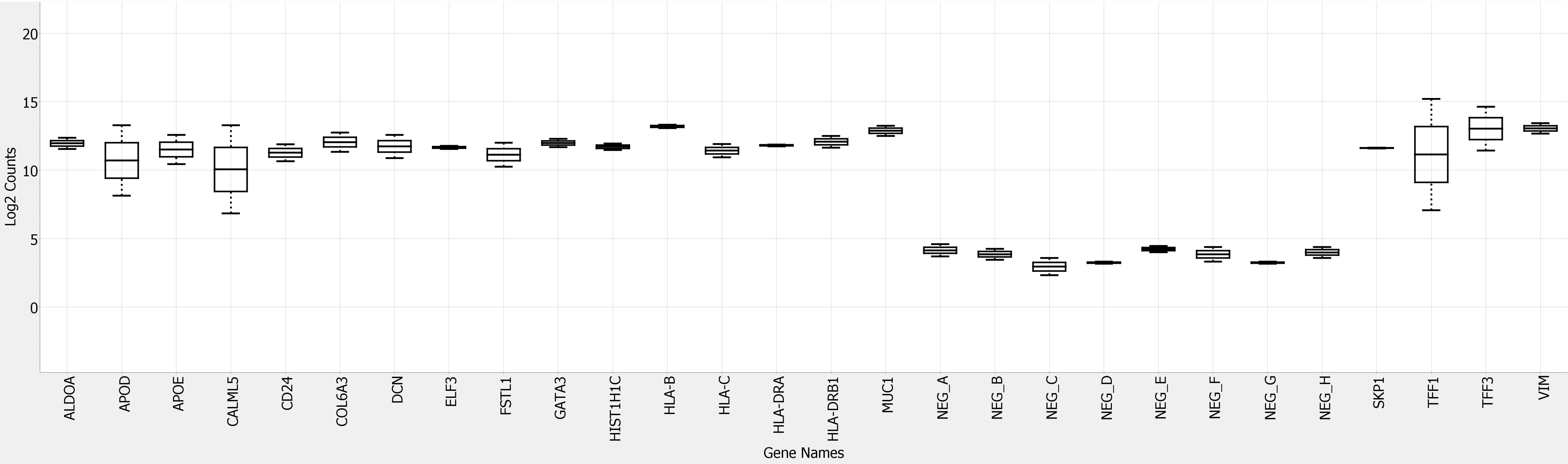

Supp. Fig. 1: Box plots showing the top 20 endogenous gene transcripts, with the highest average Log2 counts among the samples, as compared to the negative controls included in the nCounter BC360 panel. The median value is represented by a horizontal line within the box defined by the first and third quartiles. The tails represent 1.5x the interquartile range. The negative (NEG) control probe counts can determine the background threshold, thus the rate of false discovery.
